# CNN Hardware Accelerator for Real-Time Bearing Fault Diagnosis

**DOI:** 10.3390/s23135897

**Published:** 2023-06-25

**Authors:** Ching-Che Chung, Yu-Pei Liang, Hong-Jin Jiang

**Affiliations:** Department of Computer Science and Information Engineering and Advanced Institute of Manufacturing with High-Tech Innovations, National Chung Cheng University, Chia-Yi 621301, Taiwan; ypliang@cs.ccu.edu.tw (Y.-P.L.); hong@s3lab.org (H.-J.J.)

**Keywords:** fault diagnosis, convolution, neural networks, quantization, fixed-point arithmetic, real-time systems, field-programmable gate arrays, signal sampling, digital signal processing, digital circuits

## Abstract

This paper introduces a one-dimensional convolutional neural network (CNN) hardware accelerator. It is crafted to conduct real-time assessments of bearing conditions using economical hardware components, implemented on a field-programmable gate array evaluation platform, negating the necessity to transfer data to a cloud-based server. The adoption of the down-sampling technique augments the visible time span of the signal in an image, thereby enhancing the accuracy of the bearing condition diagnosis. Furthermore, the proposed method of quaternary quantization enhances precision and shrinks the memory demand for the neural network model by an impressive 89%. Provided that the current signal data sampling rate stands at 64 K samples/s, the proposed design can accomplish real-time fault diagnosis at a clock frequency of 100 MHz. Impressively, the response duration of the proposed CNN hardware system is a mere 0.28 s, with the fault diagnosis precision reaching a remarkable 96.37%.

## 1. Introduction

Recently, advanced manufacturing systems have relied on complex and precise equipment to improve efficiency and operator safety. For example, in the industrial era, large-scale manufacturing industries have needed to use many electric motors instead of laborers because these motors are easy to install and operate and have a strong overload capability, saving labor costs and avoiding human mistakes. However, the mechanical parts are easily damaged when the motor runs for a long time. Statistical analysis reveals that the mechanical failure of bearings accounts for 40% and 90% of large and small machines, respectively [1]. Moreover, the inability to find the failure of mechanical parts in time causes catastrophic chain damage to all of the equipment. In other words, how to detect failure in time is an important issue for industries, especially with regard to bearing faults.

Typically, there are two main types of bearing fault diagnosis: (1) the remaining useful life (RUL) and (2) fault category diagnosis. The RUL estimates how long the machine can operate before the bearing needs to be replaced. Meanwhile, fault category diagnosis refers to the accurate identification of the type and location of the fault. Their common goal is to enable the effective implementation of maintenance work while preventing the shutting down of the production line due to severe mechanical failures, causing high losses.

According to the observation of a previous study [2], localized faults in rolling bearings produce a series of broadband impulse responses in the vibration signal when the bearing components repeatedly strike the fault. Moreover, the exact location of the fault determines the nature of the impulse response series, and it can be observed through the power spectral density (or envelope spectrum). Therefore, Lessmeier et al. [2] indicated that each fault location has a different characteristic frequency in the frequency spectrum. 

Nevertheless, some of the previous works [3,4,5,6,7,8,9] detect the bearing faults by collecting the vibration signal and then analyzing the data to classify the fault types. In addition to the works on the detection of bearing faults, the previous work [10] indicated that the vibration signal would be influenced by the surface topography of raceways. In other words, some defects of bearing components would transfer to other components; this phenomenon is called operational heredity. Therefore, using the vibration signal for fault diagnosis may require consideration of the influence of the topography of raceways. However, due to the size of the accelerometer, high cost, inconvenience of installation, and the impossibility of dismantling some bearings, the current data are gradually being considered for fault diagnosis [1], which can be measured by installing a low-cost frequency converter or a current transformer. In other words, to avoid the troublesome installation, the magnetic effect of the current can be used to measure the stator current through the electromagnetic field generated around it. The measurement consists of a current transformer with a simple closed core and winding.

Additionally, the Mechanical Engineering Construction and Drive Technology Research data center of Paderborn University [11] offers a publicly accessible dataset for researchers. This dataset from Paderborn University (PU) includes information on fault locations, such as the inner and outer races and a combination of the outer and inner races. Note that, in the following paper, the dataset is referred to as the PU dataset for short. The damage is divided into artificial damage, such as electric discharge machining and drilling of the bearing, and real damage, which is produced by an accelerated life test, usually by applying a higher radial force than usual when the bearing is used and setting improper lubrication to accelerate the damage.

Being a rather comprehensive dataset, several studies [1,12,13,14,15,16] have attempted to develop methodologies for fault diagnosis and to categorize fault types using the current signal from this dataset. However, most of them are challenging to implement in the hardware of embedded systems due to their high computational complexity. In other words, there is no existing research on the circuit implementation of bearing fault diagnosis using this dataset. The current signal data for the bearing condition diagnosis for this dataset are measured with a low-cost current transducer. However, the current signal can be more easily masked by noise than the vibration signal when the bearing is running [17]. Therefore, finding an effective method to extract the current signal features is necessary. On the other hand, the previous works are often accompanied by high computational overhead; therefore, they cannot be integrated into the machine tool and can only be used for offline data analysis of bearing condition diagnosis.

To address the problems mentioned above, this work aims to perform a real-time condition diagnosis of bearings through low-cost hardware circuits, eliminating the need to upload data to a cloud computing server. Furthermore, implementing a hardware accelerator to perform analysis on the sensor side can be highly integrated with the machine tool to improve its self-monitoring capability. 

The main contributions of this paper can be summarized as follows:

(1) This work is a pioneer work that considers the feasibility of circuit implementation and the real-time response ability in bearing fault diagnosis problems using current signals.
(2) Furthermore, the signal used for diagnostics is the current signal rather than the vibration signal. Thus, the cost of the vibration sensor can be reduced, which is also a better solution for bearings that are difficult to disassemble. In other words, low hardware cost is achieved without losing too much accuracy.
(3) In this work, the complex data preprocessing procedures are removed without influencing the accuracy.
(4) In addition, this work converts the convolutional neural network (CNN) model from a commonly used two-dimensional CNN to a one-dimensional CNN to further reduce the delay in starting the operation. It is no longer necessary to wait for two rows of data before the convolution operation; it is only necessary to wait for kernel-sized data.
(5) On the other hand, this work proposes a new quaternary quantization method to replace the ternary quantization method. As a result, the weight of the CNN can be better expressed.
(6) Moreover, accuracy is improved without increasing the number of bits representing the weight. Therefore, the cost of the circuit implementation can be reduced without losing much accuracy.
(7) The procedure for realizing hardware circuits is introduced in this paper.
(8) The experimental results show that the proposed method can achieve similar accuracy to that of previous works on model accuracy with a significantly lower hardware cost.

The remainder of the paper is organized as follows: Section 2 introduces the related work on bearing fault diagnosis. Section 3 shows the proposed methodology of the bearing fault diagnosis and the CNN architecture in both the software and the hardware implementation. Then, the experimental results are discussed in Section 4. Finally, the conclusion is given in Section 5.

## 2. Related Work

Over the decades, different optimization methods and models of different architectures were proposed by different parties for powerful classification tasks. On the other hand, in the field of bearing faults diagnosis, the traditional method requires a thorough understanding of the mechanism of the machine. However, as the machine becomes more complex over time, the external environment’s noise makes the traditional methods inefficient and difficult to apply. Therefore, research on fault diagnosis has gradually shifted from signal analysis to autonomous neural network training models, where the training network model does not require prior domain knowledge. Moreover, fault diagnosis can be considered as the feature extraction and classification problem; the autoencoder (AE), deep belief network (DBN), support vector machine (SVM), and CNN are commonly used machine learning methods for feature extraction and classification. Therefore, several types of research aimed to apply those methods to the bearing diagnosis area.

For example, Qin et al. [3] proposed an optimized DBN model and a logistic sigmoid function that solves the vanishing gradient problem of the sigmoid function to improve the accuracy of the DBN in fault diagnosis. This research also proposed a method to preprocess the network input signal by first applying the Morlet wavelet transform to the original vibration signal, using the kurtosis index to select impulsive components and an adaptive soft-thresholding method to reconstruct the signal. The proposed method is better than the empirical mode decomposition for the extraction of signal features. However, the problem of the network optimization of the DBN still exists, making practical applications of the DBN difficult to achieve in the industry. 

On the other hand, Abid et al. [18] used the SVM method of the directed acyclic graph (DAG) to classify faulty bearings and improved the performance of the SVM by combining the stationary wavelet packet transform (SWPT) and the DAG. The current signal was used, and the SWPT was applied to extract features and to overcome the shortcomings of other wavelet transforms. Furthermore, Wen et al. [19] used the classical CNN, LeNet-5, and proposed a method for signal image conversion. Wen’s work [19] randomly took the segment of the vibration signal rather than the whole data in one record. These signals were then converted into a two-dimensional image with a size of 64 × 64, which was inputted into the CNN model. Their research has inspired a deeper investigation into the use of CNNs for fault diagnosis. Previously, inputs based on a frequency domain or complex feature extraction caused application difficulties. The CNN model based on the time-domain signal enables the realization of a real-time diagnosis method. 

More recently, Karpat et al. [15] used a one-dimensional CNN with a large window size for classifying bearing faults. However, the proposed CNN model requires over 1422k parameters and only achieves an accuracy lower than 95%. Furthermore, Zhu et al. [4] preprocessed the raw vibration sensor data to extract nine features for the time, frequency, and time–frequency domains. A CNN network was then applied for machine health monitoring. However, the proposed method with complex preprocessing is only suitable for offline analysis applications. Moreover, Tan et al. [5] tried to reduce the amount of training datasets with a few real damaged data and proposed two deep coupled CNN networks for extracting features from the raw data. However, only the data of the artificial and real damage acquired on an identical machine were tested. 

Magar et al. [6] extracted 14 features from raw signals to reduce the preprocessing overhead. However, their approach is computationally expensive and may not be suitable for real-time fault classification. Moreover, Xu et al. [8] proposed the TCNN, which can achieve an online fault diagnosis. TCNN is based on LeNet-5 and an offline auxiliary CNN. Although the work achieved the online analysis, it used vibration signals as the input of the model, which had a relatively high-cost (compared to the current signal), and all the computation had to be conducted on an external server.

On the other hand, a few-shot learning method [7] was proposed to leverage past data to learn new tasks quickly for fault classification. However, only 13 of the total 32 representative classes were tested. On the other hand, Hou et al. [20] proposed a signal-to-input feature mapping to convert raw data into images. However, current and vibration signals are required to improve the fault classification accuracy in their approach. For real damage data generation, a one-dimensional generative adversarial network [21] was proposed. However, the testing accuracy was lower than 70% when the CNN network trained with real data samples was used for classifying the generated data samples. 

Wang et al. [22] used small and large convolution kernels for feature extraction. However, the proposed method requires the fault frequency to determine the suitable size of the kernel, or the classification accuracy could be lower than 70%. A lightweight and efficient feature extraction network was presented in [23] to reduce the number of network parameters. However, the accuracy was less than 95% in all cases.

Moreover, Sabir et al. [16] suggested a method for bearing fault diagnosis based on long short-term memory (LSTM). Eight features were extracted from raw signals, a computation-intensive process, making it unsuitable for real-time fault classification. Conversely, a recent study [9], which was mindful of the computational overhead, introduced a lightweight convolutional neural network (CNN) model for fault diagnosis that was applicable to edge AI devices; the PU dataset was utilized for the research. However, their model only incorporates vibration signals. As previously stated, such signals may not be appropriate for certain bearings that cannot be disassembled, potentially limiting this work’s applicability. Finally, Hoang et al. [12] highlighted the challenges of using a current signal as the input to train a CNN model. They proposed a deep neural network (DNN)-based model with information fusion to improve model accuracy. Even though this approach can yield high accuracy, the hardware cost was not considered in this work. Therefore, this method is not suitable for implementation in a hardware circuit.

As discussed above, many studies have shown that using a neural network for fault detection is more accurate and stable than conventional methods and requires less computation time [3,19]. In the diagnosis of rolling bearing faults, it is also believed that some common model-based methods, such as the SVM or k-nearest neighbor algorithm (KNN), are unable to effectively extract and eliminate higher-dimensional features during feature extraction [2]. Furthermore, when the training data are time-domain signals, CNN has been more accurate than other model-based methods, such as the multilayer perceptron (MLP) and AE [24]. However, input data preprocessing is too complicated for many of the above networks for real-time diagnostic analysis. For example, input preprocessing using fast Fourier or wavelet transform requires powerful hardware support to perform real-time diagnostic analysis. To sum up, to the best of our knowledge none of the previous works can achieve real-time analyzing requirements given the hardware implementation costs.

## 3. Methodology

### 3.1. Overview of the Proposed Method

**Figure 1:**
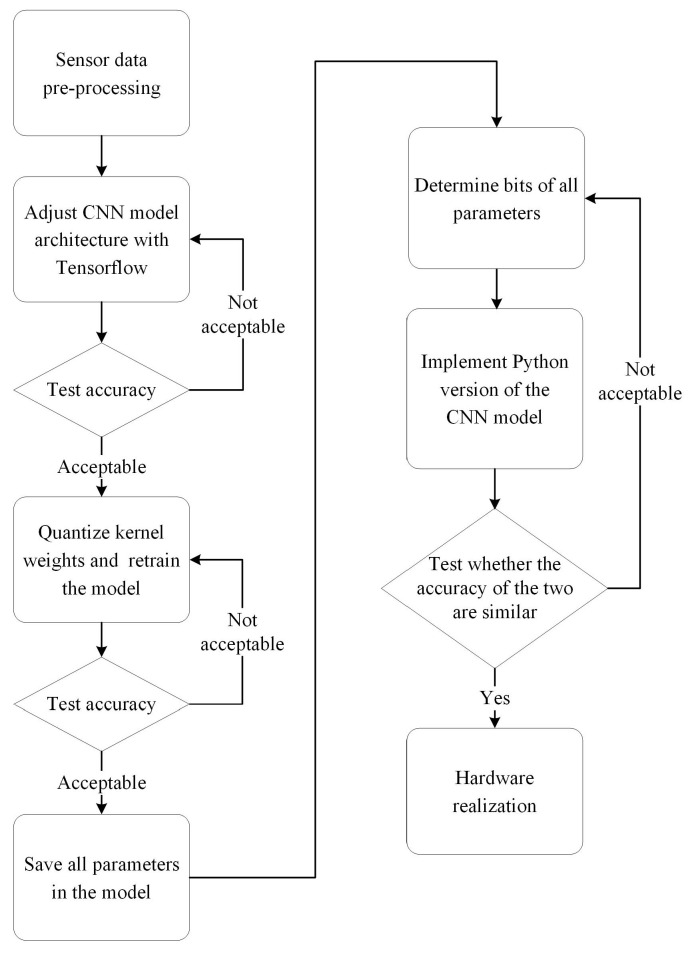
The flowchart of the proposed design method.

As previously stated, this work aims to propose a CNN-based method for bearing fault diagnosis that achieves high accuracy, low hardware implementation costs, and real-time responsiveness. Figure 1 presents the flowchart of the proposed design steps. As depicted in Figure 1, the main design steps can be broadly separated into software and hardware phases. The proposed CNN model was adjusted during the software phase to meet the design objectives. Initially, a data preprocessing flow was used to gather data from sensors to serve as input images for the CNN model. Following this, a sequence of experiments on the TensorFlow platform using Python was conducted to adjust the CNN model. Note that all the experiments in this step were performed on the TensorFlow platform with the hyperparameters shown in Table 1, and the input data were sourced from the current data in the PU dataset [11]. Additionally, all the analyses and discussions in this paper were based on the PU dataset.

In the step of adjusting the CNN model, many aspects and parameters had to be determined and tested, such as the CNN model architecture and the weight quantization, among others. Every detail in the proposed design was decided upon through theoretical assumptions and experimental testing. The proposed model’s accuracy was tested after each step, and if the accuracy met the expectations, then the next step was carried out. Once all the CNN model details were finalized, the last software stage step was to develop a Python program for the final CNN model and to verify its functionality and accuracy compared to the TensorFlow version. The proposed model’s algorithms could be validated and implemented into a hardware unit by developing the Python version of the CNN model.

Finally, the design moved to the hardware implementation phase, where the register transfer level (RTL) Verilog code for the proposed CNN model was created to develop the proposed method in the hardware. Once the hardware design was completed, the proposed method was evaluated on an FPGA to verify the proposed method’s capabilities and functionalities. The details of each step in this flowchart are explained in the subsequent subsections.

### 3.2. The Proposed CNN Architecture

#### 3.2.1. Bearing Data Preprocessing

This paper used the bearing dataset of PU [11] to train the CNN model for the analysis of the bearing condition. Two-phase current signals were measured by a current transducer and converted to a digital signal for storage using a 25 KHz low-pass filter and an analog-to-digital converter with a sampling rate of 64 KHz. There were four types of classification results, and the amount of data in each category was not the same. As shown in Table 2, one volume of data represents a collection of 80 records with a length of 4 s. Thus, for example, label 0 has 480 (6×80) records. In Table 2, the outer race fault (label 1) indicates damage occurring on the bearing’s outer race. Similarly, inner race faults (label 2) signify damage to the bearing’s inner race. Conversely, the third fault type (label 3) corresponds to a situation where damage occurs on both the inner and outer race of the bearing. Finally, if the bearing operates normally, the bearing is considered as the health status with label 0.

**Figure 2:**
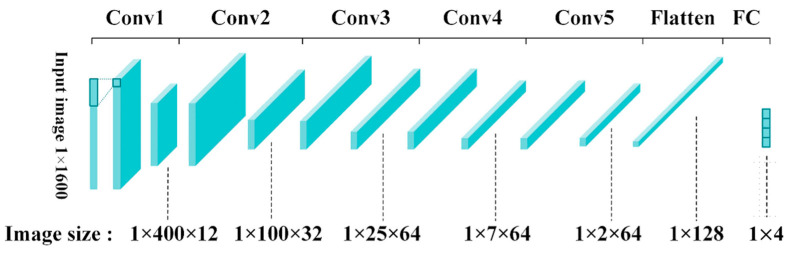
The input image size of the proposed one-dimensional CNN in each layer.

More specifically, for the training and testing of the model, all the data in the dataset were randomly divided into 80% training data and 20% test data during the CNN training process. Note that the test data were not allowed to participate in the training process but were only used in the inference phase to test the accuracy of the CNN model. Figure 2 shows the overview of the processing flow of the proposed one-dimensional (1-D) CNN network with an input image size of 1 × 1600. In more detail, a conv block contains a one-dimensional convolution, batch normalization function, and pooling operation in the proposed method. Notably, the image size shown in Figure 2 represents the size of the feature map after each stage. To provide a better understanding of the proposed model, the following paragraphs introduce the detail of the model and the reasons for selecting the parameters.

First, the input image’s size is a critical factor in CNN model design. The primary consideration in determining the input image size is the relationship between the length of the observation time (i.e., the time interval for collecting current data) and the accuracy. Generally, a longer observation time may yield higher accuracy in the context of bearing fault diagnosis. However, extended observation time leads to a larger input image due to an increase in the number of observation points. As the image size expands, the associated hardware costs also rise. Consequently, this work selected an appropriate image size that balanced accuracy and hardware implementation costs.

As mentioned earlier, the challenge when deciding on the image size can initially be simplified to the determination of the observation time. Theoretically, if a bearing fault occurs, it will be detected periodically due to the rotational nature of the bearing. Moreover, the frequency at which the fault is identified may align with the ball-passing frequency. Hence, studying the ball-passing frequency assists in the determination of the observation time. 

The ball-passing frequency can be divided into outer race ball-passing frequency (abbreviated as *F_o_*) and inner race ball-passing frequency (abbreviated as *F_i_*). The theoretical formula for these two types of ball-passing frequencies is illustrated in Equations (1) and (2) [25], where *F_r_*, *n*, *d*, *D*, and *α*, represent the rotary frequency (Hz), number of rolling elements, ball diameter, pitch diameter, and bearing contact angle between the ball and the cage, respectively.

Meanwhile, the dataset used in this study (i.e., the PU dataset [11]) offers data at two rotation speeds: 900 rpm and 1500 rpm. Consequently, with Equations (1) and (2), the ball-passing frequencies for these two rotation speeds can be calculated; they are listed in Table 3. As depicted in Table 3, the lowest frequency for the inner and outer races is 45.7 Hz. That is to say, once a fault is detected, the fault will manifest at least once every 21 ms. Thus, the observation time should be set to a minimum of 21 ms to detect all the types of faults in the PU dataset.

(1) $$F_{o}=\frac{n\cdot F_{r}}{2}\cdot \left\{\;1-\frac{d}{D}\;\cdot \;\cos\alpha \;\right\}$$

(2) $$F_{i}=\frac{n\cdot F_{r}}{2}\cdot \left\{\;1+\frac{d}{D}\;\cdot \;\cos\alpha \;\right\}$$

A series of experiments were conducted to validate the assumption mentioned above. The CNN models were retrained with various input image sizes (observation times) and compared to their accuracy in these experiments. Table 4 presents the accuracy results for different input image sizes. Note that since the sample rate of the dataset used is fixed (i.e., 64 K samples/s), the input image size can be directly calculated by multiplying the sample rate by the observation time. Therefore, the input image size can be determined as well. As shown in Table 4, if an image contains 625 data points, the length of the signal that can be observed is 9.7 ms. If the time length corresponding to the sensor data contained in the image is insufficient to identify the fault feature, the accuracy of the trained model is considered insufficient. Table 4 shows that when the image is enlarged to 1600 data points, a signal time of 25 ms can be seen in an image, corresponding to the cycle time of all ball-passing frequencies, and the model’s accuracy can be increased to 96.5%. Considering that the input image size is proportional to the required random access memory (RAM) and the best accuracy, the input image size was set to 1 × 1600.

**Figure 3:**
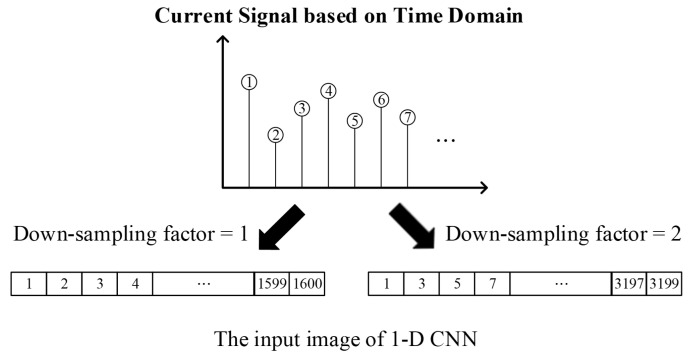
The diagram of the down-sampling process with two examples of the down-sampling factor.

Once the input image size is determined, given the fixed sampling rate, the observation time also becomes fixed (i.e., 25 ms in the PU dataset). However, it is typically observed that longer observation times can enhance noise tolerance ability and further boost fault detection accuracy. Therefore, with the image size fixed, a down-sampling process was conducted to extend the observation time length; this is a frequently utilized method for noise filtering and real-time processing. Figure 3 shows the process of down-sampling, which samples the original signal at intervals with the specified down-sampling factor. For example, when the down-sampling factor is 1, the original data are considered as output. On the other hand, when the down-sampling factor value is 2, each sample skips the next data point.

Moreover, in this paper, the down-sampling method was applied to increase the time length of the signal that can be viewed in an image and to diagnose the bearing condition more accurately. Therefore, the intermediate points were not discarded during the down-sampling process in the network training. As shown in Figure 3, for an image with a down-sampling factor of 2, sample points with even index numbers are discarded. However, these data with even index numbers will also produce an image for the training of the CNN model. Therefore, the total number of images for training the CNN model does not change.

**Figure 4:**
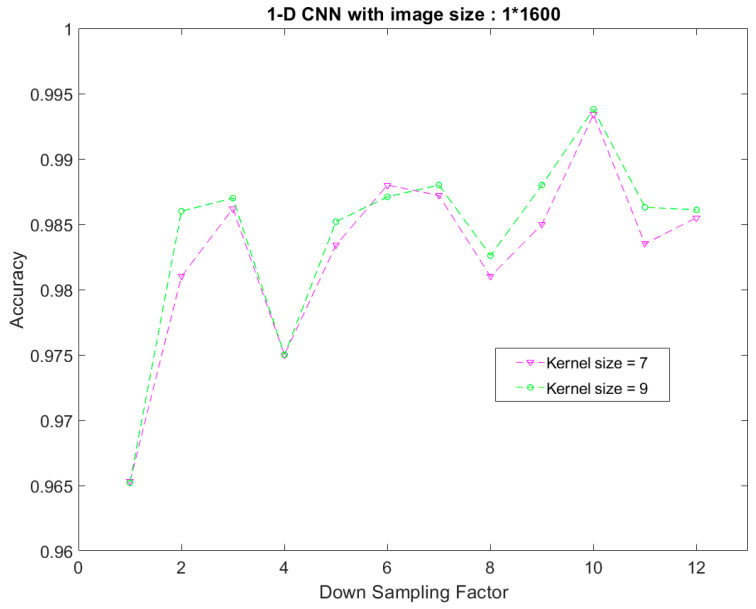
The comparison of the model accuracies obtained by different down-sampling factors.

Figure 4 compares the model accuracy measured with different down-sampling factors for processing the input data to investigate the effect of the down-sampling process. Note that the size of the input image and the size of the CNN model were the same in each experiment. Furthermore, two kernel sizes (i.e., 7 and 9) were used to observe the kernel size’s influence on the accuracy of the proposed CNN model. As shown in Figure 4, the accuracy of the CNN model can be further improved when the down-sampling method is used. For more detail, the best accuracy was achieved when the down-sampling factor was 10, reaching 99.34% and 99.38% for kernel sizes 7 and 9, respectively. 

On the other hand, regarding the kernel size selection, a smaller kernel size saves memory usage for the same network structure but may reduce model accuracy. As shown in Figure 4, the difference in the model’s accuracy between two different kernel sizes at a down-sampling factor of 10 is only 0.04%, but using the smaller kernel can save 22% (i.e., $\frac{\left(9-7\right)}{9}\times 100\%$) of the weight memory usage. Therefore, with the hardware implementation cost consideration, this paper used a down-sampling factor of 10 and a kernel size of 7 for the preprocessing procedure of CNN model training.

Furthermore, in CNN model training, overlapping images can usually further improve the accuracy of the CNN model. This method has a good effect on neural networks with a small training dataset. The overlapping method means that after sampling the signal in the first image, the sampling position of the second image starts from the specific position of the first image and so on. Generally, the overlap is about 25% of the image. However, it should be noted that images used by the overlapping method are only used for the training and validation dataset and not for the test dataset. Otherwise, the test dataset may not be independent of the training dataset.

**Figure 5:**
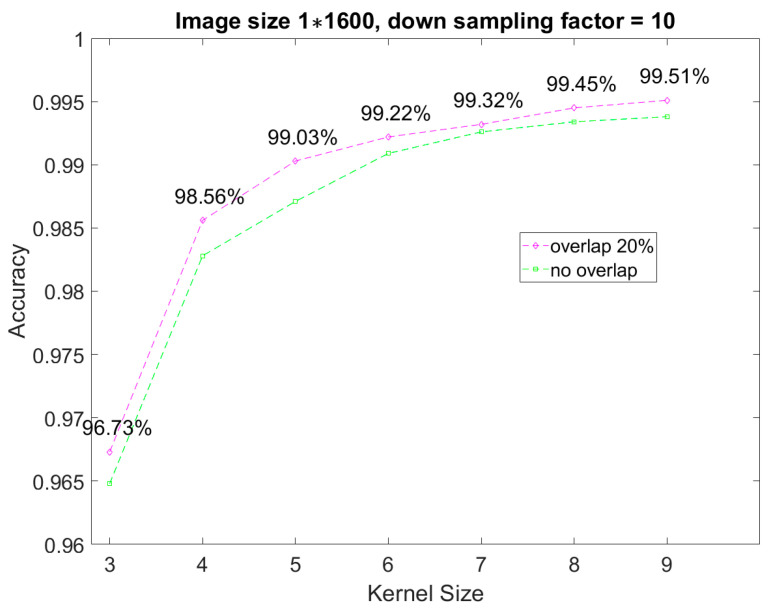
The model accuracies were compared by different kernel sizes and overlap percentages.

Figure 5 compares the model accuracy trend as the kernel size changes using 20% of the image data overlap, or when no image data overlap is used. The percentage marked on the graph is the model’s accuracy using 20% of the image data overlap. The accuracy of the CNN model shown in Figure 5 is the average of multiple repeated trainings. Under different kernel sizes, the model’s accuracy was improved by using 20% of the image data overlap. When the original sensor signal is cut into different images, the characteristic signal may be cut into different images and ignored. The 20% image data overlap can reduce the chance of feature signals being segmented. Therefore, this paper used a 20% overlap of the training data in the software training, which was randomly selected for each training.

#### 3.2.2. CNN Architecture and Quaternary Quantization

After introducing the bearing data preprocessing procedure and related parameters, this subsection shows the proposed CNN architecture and quaternary quantization method. Table 5 shows the proposed CNN model architecture. First, a conv layer contains zero paddings, convolution, ReLU function, and batch normalization operation, followed by a max-pooling layer with a size of 1 × 4. Although performing the max-pooling operation, the input image’s size may not be a multiple of 4. At this time, zero values are added to the boundary of the image to avoid losing features. After the last max-pooling operation, the output image is flattened to a size of 1 × 128 and sent to the fully connected (FC) layer. Finally, the calculation results of the four categories are obtained, and the category with the largest value will be the network classification result.

On the other hand, when calculating the CNN network, the parameters and input values are multiplied accordingly to extract various features. However, complicated network calculations lead to a long inference time. Furthermore, due to limited hardware resources and capabilities in mobile devices and application-specified integrated circuits that can be integrated into machine tools, the weight parameters of the network must be quantized. Therefore, a tradeoff must be made between the model accuracy and the hardware complexity. Generally, a quantization technique can reduce the required memory space, the number of bits needed for computation, and the circuit’s power consumption and simplify the hardware design’s complexity. Various quantization methods have been adopted in previous works [26,27]; therefore, it is necessary to further investigate which quantization method is the most suitable for the proposed model.

To understand the effect of each quantization method on the proposed CNN model, an experiment was conducted to evaluate the capability of three quantization methods adopted to the proposed model. Table 6 shows the results of the accuracy influenced by the different quantization methods. Note that all the experiments used the PU dataset in this work and that the settings of the CNN model were also identical among all the experiments. In more detail, the binary quantization [26] is a 1-bit quantization. This means that the converted weight has only two possible values, −1 or +1, which facilitates the implementation of a lightweight network. The memory requirement for the weight or activation value is only 1 bit and does not take too much memory.

On the other hand, the ternary quantization [27] method converts the weight from a 32-bit full-precision representation into three values. Li et al. [27] proposed this method. Initially, the weights are replaced by {−1, 0, 1}. However, to reduce the loss of accuracy by using only {−1, 0, 1}, ref. [28] suggested replacing ±1 with floating-point values. The ternary floating quantization process must divide all weights by the maximum weight to map the weights into [−1, +1]. All layers then use the same threshold (±0.05) to classify the weights numerically as {−1, 0, +1}. In the scaling phase, −1 is multiplied by *W_n_* and +1 by *W_p_*. *W_p_* and *W_n_* are parameters that are trained jointly with the CNN model. Different layers of *W_p_* and *W_n_* are trained separately and have a special formula to calculate the gradient. Finally, all the weights can be expressed by {−*W_n_*, 0, *W_p_*}. 

As shown in Table 6, the accuracy of the proposed CNN model after quantization was insufficient. There are two commonly used methods to improve the accuracy, including (1) increasing the number of channels of each convolution layer and (2) improving the quantization method. However, the first method increases the memory and computation requirements and is therefore unsuitable for hardware with limited memory space. Therefore, in this paper, an improved quantization method is presented.

In the previous works [27,28], the threshold values were set to ±0.05 for the ternary quantization with {−1, 0, 1} integer weights. Thus, a trial test was performed by changing the threshold value of the ternary quantization to find the best floating weight for the proposed model. As a result, the threshold was no longer a fixed value but was calculated dynamically with the weight distribution of each layer. Equation (3) defines the procedure to set the threshold, where mean (*W*) and *σ* represent the mean and standard deviation of the weights in each layer, respectively. Moreover, *x* in the equation is a variable that varies the threshold in the trial test. Note that each layer can have its threshold value.

(3) $$w^{t}=\{\begin{array}{c} W_{p}\;:\;W\geq Mean\left(W\right)+x\cdot \sigma \;\;\;\;\;\;\;\;\;\;\;\;\;\;\;\;\;\;\;\;\;\;\;\;\;\;\;\;\;\;\;\;\;\;\;\;\;\;\;\;\;\\ 0\;:\;Mean\left(W\right)-x\cdot \sigma <W<Mean\left(W\right)+x\cdot \sigma \\ -W_{n}\;:\;W\leq \;Mean\left(W\right)-x\cdot \sigma \;\;\;\;\;\;\;\;\;\;\;\;\;\;\;\;\;\;\;\;\;\;\;\;\;\;\;\;\;\;\;\;\;\;\;\;\;\;\;\;\; \end{array}$$

**Figure 6:**
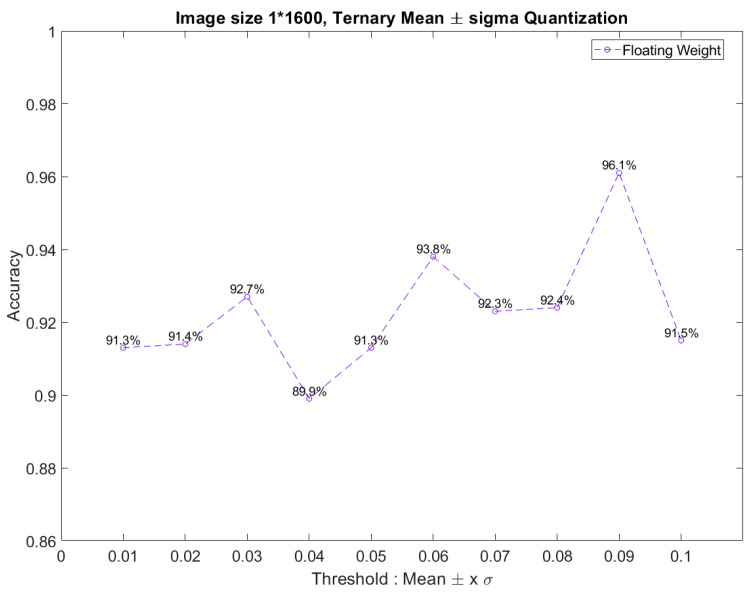
The trend of the model accuracy using the method of determining the threshold value according to the weight distribution of each layer.

Figure 6 shows the result of the trial test mentioned above. The *x*-axis in Figure 6 represents the different floating weights, and the *y*-axis denotes the variations in accuracy. As shown in Figure 6, by changing the value of *x* in Equation (3), the accuracy is improved by up to 6% compared with that of the fixed threshold method. Furthermore, the best accuracy of 96.1% was achieved when *x* was 0.09 in our model. Therefore, the method of dynamically changing the threshold value when *x* is 0.09 will be adopted.

Furthermore, when using the function provided by Tensorflow to train the neural network model to obtain weights, the weights of each trained layer will be close to the normal distribution. Table 7 shows the mean value of the weights of each layer before quantization in the four tests. Moreover, the quantization methods used in this paper were for quantization-aware training. Compared with post-training quantization, quantization-aware training can gradually adjust the model to acceptable accuracy. However, the average value of each layer was negative in most cases in the results of these four tests. Therefore, this paper proposes a new quaternary quantization method to express the negative value of weight better and to retain the weights’ two-bit representation.

In this paper, a new negative weight value of −1 was inserted into the ternary quantization to prevent the negative values near 0 from being quantized to 0 and to increase the proportion of the negative weight. More specifically, Equation (4) defines the quaternary quantization method proposed in this paper. Notably, if the −*W_n_* of a layer is not less than −1, this means that the layer has only three weights.

(4) $$w^{t}=\{\begin{array}{c} W_{p}\;:\;W\geq Mean\left(W\right)+x\cdot \sigma \;\;\;\;\;\;\;\;\;\;\;\;\;\;\;\;\;\;\;\;\;\;\;\;\;\;\;\;\;\;\;\;\;\;\;\;\;\;\;\;\;\;\;\\ 0\;:\;Mean\left(W\right)-x\cdot \sigma <W<Mean\left(W\right)+x\cdot \sigma \\ \;\;-1\;:\;W_{n}<-1\;and-1\geq W>Mean\left(W\right)-x\cdot \sigma \;\;\;\;\;\;\;\\ -W_{n}\;:\;W\leq \;Mean\left(W\right)-x\cdot \sigma \;\;\;\;\;\;\;\;\;\;\;\;\;\;\;\;\;\;\;\;\;\;\;\;\;\;\;\;\;\;\;\;\;\;\;\;\;\;\;\;\;\;\;\;\;\; \end{array}$$

**Figure 7:**
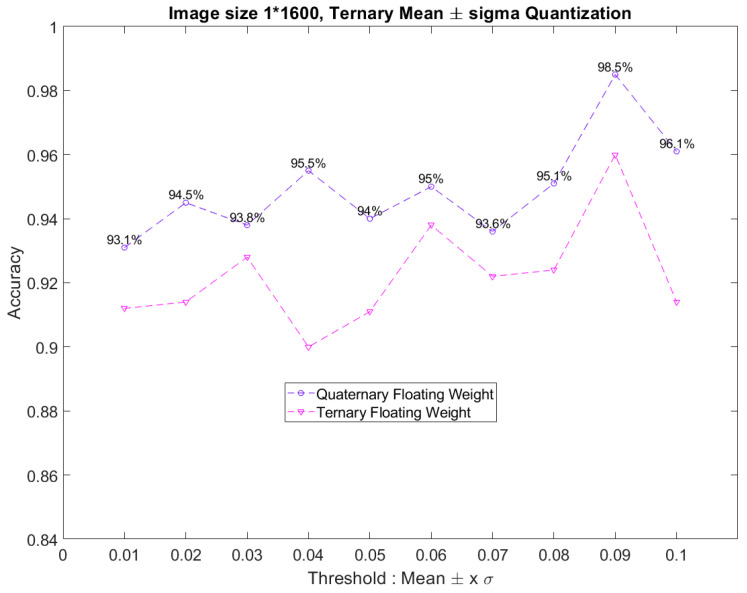
The comparison diagram of model accuracies obtained by the dynamic threshold with ternary and quaternary quantization.

With the proposed quantization methods, Figure 7 shows the accuracy based on the dynamic threshold with ternary and quaternary quantization. The weights *W_p_* and *W_n_* of the two methods are both floating-point numbers. As Figure 7 shows, the accuracy of the proposed quaternary quantization outperforms that of the ternary quantization for different thresholds. Remarkably, the most appropriate threshold value with the highest accuracy is still when *x* is 0.09, and the accuracy can be increased from 96.1% to 98.5%. However, the value of *x* should not be too large. Thus, the number 0.09 was adopted for *x*. Because of the normal distribution of weights, many weights become 0 after quantization, and the accuracy decreases. Therefore, this paper used the quantization method of weights in Tensorflow training, using the quaternary quantization of the dynamic threshold with floating-point numbers.

When the weight quantization method is determined, it is necessary to reduce the number of channels in each layer to reduce the number of parameters of the network model and the cost of hardware implementation. The relationship between the number of channels and the accuracy should be investigated first to choose the number of channels. Therefore, an experiment was conducted to obtain accuracy with different settings of the number of channels. Table 8 shows the results of the experiment. In Table 8, C_1_ to C_5_ represents the number of channels in the proposed CNN model’s first to fifth layers. On the other hand, Table 8 also shows the number of parameters corresponding to each version of the settings.

In the previous design experience, a CNN model had to have enough output channels for each layer; however, too many output channels also increased the memory and computation time. In observations of the experimental results, the first and second layers play an essential role in filtering noise in a CNN model. Therefore, the number of output channels of C_1_ and C_2_ cannot be too small. Otherwise, it is difficult to improve the accuracy of the model. Furthermore, the experimental results also show that increasing the number of output channels layer by layer is the best method. 

In terms of hardware implementation, the goal is to minimize the memory requirements. Therefore, the best practice is to use ping-pong RAMs to store the computed feature maps for each layer. Moreover, the size of these two RAMs is usually determined by the largest feature map that needs to be stored. Because the feature map size will become smaller after the pooling operation, the largest feature map is usually the output of the first and second layers. Therefore, if the number of output channels of C_1_ and C_2_ increases, the ping-pong RAM size will increase accordingly.

To summarize the considerations and observations above, the number of output channels in C_1_ and C_2_ of versions 3 and 4 was better for hardware implementation, but the accuracy was unacceptable, as shown in Table 8. The model’s accuracy could be improved by increasing the number of C_2_ output channels in versions 3 and 4, which became versions 5 and 6. Version 7 was an extension of version 6 with only the number of C_1_ output channels increased, and the number of parameters did not increase excessively. However, the size of one of the ping-pong RAMs had to be increased by 25%, but the model’s accuracy was improved by only 0.2%. This tradeoff is not cost-effective. Therefore, this work finally adopts the number of channels of version 6 to determine the final CNN architecture used.

**Figure 8:**
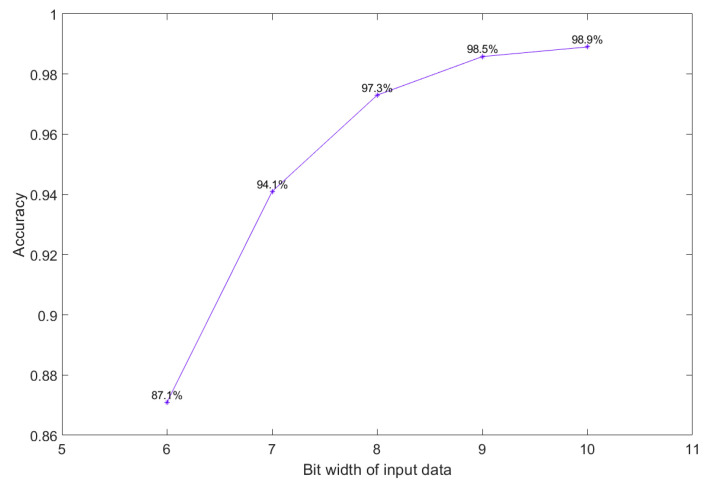
Accuracy trend with a different bit width of the input data.

During the training process in the software stage, a 32-bit floating-point operation is often used. However, the considerable cost of floating-point operations is unacceptable for applications with limited hardware and power consumption. Therefore, the input signal and the trained parameters must be quantized. In other words, a tradeoff must be made between the accuracy and the limited computational resources. To avoid decreasing accuracy significantly, an experiment was conducted to obtain the accuracy trend with different bit widths of the input data, and the results are shown in Figure 8. Note that, in this experiment, to fully express the integer digits 4 bits must be used for the integer part. The unnecessary decimal bits can be removed by a simple signal preprocessing method. The accuracy was 99.3% when using raw signals for training. If the decimal bit was 5 bits, the model accuracy decreased only 0.4% compared to 6 bits, and memory space could be saved. Therefore, 4 integer bits and 5 decimal bits were used for the input data.

On the other hand, speaking of the bit width of the weight value, according to the proposed quaternary quantization method only 2 bits are needed to store a weight value in the read-only memory (ROM). However, a lookup table corresponding to the actual weight value must be set up. At the same time, the integer bits and decimal bits must also be specified for these actual weight values because the convolution operation multiplies the weight by the input value. In addition, multibit multipliers and adders cause bottlenecks in the circuit speed and significantly increase power consumption.

Similarly, to select the proper number of bits used in the kernel table, an experiment was also conducted, and a table was built to understand how the accuracy was influenced by different settings. Table 9 shows the test by adjusting the number of decimal bits after fixing the integer bits to 5 bits. Considering the accuracy objective, which was set at 98%, and the cost of the multipliers and adders, the weight bit width of 14 bits was selected, including 5-bit integers and 9-bit decimals.

Moreover, since the multiplication and accumulation process of the convolution layer takes a lot of time, two RAMs were used as ping-pong RAMs for the intermediate storage of the feature map values. Until all the calculations of the current layer were completed, these maps were read out as the input for the next layer. Therefore, the bit width of the map had to be set to avoid massive memory space. 

Therefore, the relationship between the feature map bit number and the accuracy was also examined by an experiment. Table 10 shows the model accuracy results of limiting the bit number of feature maps. After testing all the patterns, the simulated extreme values of the feature maps in each layer were 113 and −34. Therefore, 8 bits were required for the integer bits. Subsequently, several tests were performed to determine the number of decimal bits. When the decimal bits increased from 8 bits to 9 bits, the improvement in model accuracy was minimal. Therefore, bits of the feature map were set to 8-bit integer bits and 8-bit decimal bits.

### 3.3. Hardware Implementation

After all the parameters in the CNN model and feature map values that needed to be stored in the memory were expressed in fixed-point numbers, the hardware description language was used to implement the proposed CNN hardware design. In addition, the accuracy and performance of the CNN circuit were also tested on the field-programmable gate array (FPGA). 

**Figure 9:**
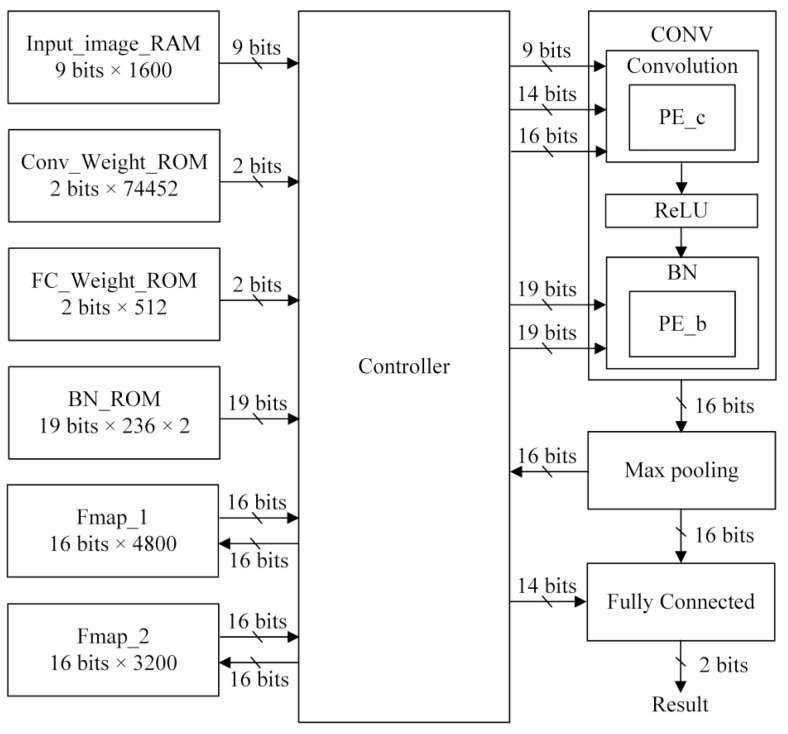
The overall architecture of the proposed CNN hardware design.

Figure 9 shows the overall block diagram of the proposed CNN hardware design. After preprocessing the PU dataset, it is converted into numerous 1 × 1600 images to train and test the proposed CNN model. One image needs to be stored in the Input_image_RAM before the CNN hardware circuit starts the computation. The controller continuously fetches data from the Input_image_RAM and adds zero paddings. In each clock cycle, the controller reads the 2-bit weight from Conv_Weight_ROM. The controller obtains the actual 14-bit weight value from the lookup tables of each layer. The quaternary quantization is applied to the weights, and thus, the actual weight value of each layer is at most four values. The controller then sends the input data and the actual weight to the CONV block. The CONV block performs a one-dimensional convolution, an activation function (ReLU), and a batch normalization (BN) operation.

The processing element, PE_c, performs a multiplication operation on the inputs and weights, subsequently accumulating these results. The outcomes are then stored in the registers. The multiply–accumulate (MAC) operation result enters the ReLU block until this register has accumulated seven multiplication values. In the ReLU block, if the MAC result’s most significant bit is 1, the output becomes 0; otherwise, the output is the MAC result. The output of the ReLU block is sent to the BN block. As the batch normalization operation requires *γ* and *β*, the BN_ROM reads two 19-bit values in succession. The output of the ReLU block is multiplied by *γ*, then plus *β* by PE_b. The result of the BN operation is 16 bits.

In the max-pooling block, 1 × 4 max-pooling operations are performed after the BN block sends four values. The largest of them will be stored in Fmap_1, one of the ping-pong RAM blocks. If the layer number is 2, the input reads feature maps from Fmap_1. The following process is the same as when the layer number is 1, but the result of the max pooling is stored in another ping-pong RAM, Fmap_2. Therefore, there is no conflict between the memory required for the previous layer’s output and the output of the current layer, and lower memory usage can be achieved.

In the implemented CNN hardware design, the total memory allocation is shown in Table 11. Moreover, the memory allocation is compared to the design using 32-bit floating-point numbers. Using the proposed quaternary quantization method for kernel weights and FC weights, only 2 bits are required to store a single value in ROM, resulting in a percentage memory reduction of 93.75%.

The accuracy test reduces the number of integer and decimal bits of the BN parameters as much as possible. Therefore, the ROM memory can be reduced by 40.62%. Furthermore, the word size of the two ping-pong RAMs, Fmap_1 and Fmap_2, can be reduced from 32 bits to 16 bits, which is a 50% reduction. Adding the total number of bits from ROM and RAM, the proposed design can achieve a total memory reduction of 89.25%. The final reduction result shows that the quantization has a significant impact on reducing the memory space requirement.

Table 12 shows the accuracy of testing each label with the Python and RTL codes. The model accuracy in each label is similar. For example, the test data from Label 1 are the best, and those of Label 2 are the worst. In sum, the accuracy of the Python and RTL codes is almost the same.

## 4. Experimental Results

The capability of the proposed method was evaluated by conducting a series of experiments. To begin with, the model accuracy, architecture, and data preprocessing methods at the software level were compared with those of earlier studies. In this experiment, comparisons were made with four previous works, with the experiment being carried out on the TensorFlow platform. The hyperparameters, architectures, and parameters were established in line with the discussions in the previous sections. Furthermore, the hardware circuit design in the proposed work was implemented on a Xilinx FPGA, with the ensuing results being analyzed in this section. The section wraps up with a discussion on real-time response ability, reinforcing the fact that the proposed method indeed possesses the property of real-time computing.

Table 13 shows the software-level comparison table with the prior works using current data in the PU bearing dataset. Compared with [1,14], the proposed design has higher model accuracy. In addition, the final classification results of [1,14] were obtained using the MLP or SVM, which may cause additional computing overhead. In addition, the input used in [14] is the feature after FFT conversion, which is only suitable for offline analysis. In summary, the proposed method is relatively simple and achieves higher model accuracy than these two previous works. Note that, in the results of [1], the accuracy of training using vibration signals is 99.4% because training using vibration signals is still a bit higher than using current signals. Nevertheless, compared with this work, the accuracy difference between the proposed method and the vibration version in [1] is only 0.8%, and current signals can bring additional benefits (they are especially easy to apply to real machine tools).

Moreover, as shown in Table 13, ref. [12] has higher accuracy than the proposed work. However, the input data of [12] required normalization during preprocessing; therefore, an additional preprocessing procedure was required, making it hard to achieve real-time response ability. Moreover, in [13], the long-term, short-term memory (LSTM) model is added after the convolutional layer, and the KNN model is added after the softmax function as the final classifier; so, it has higher accuracy. However, due to the high computing complexity, ref. [13] is unsuitable for real-time computing.

**Figure 10:**
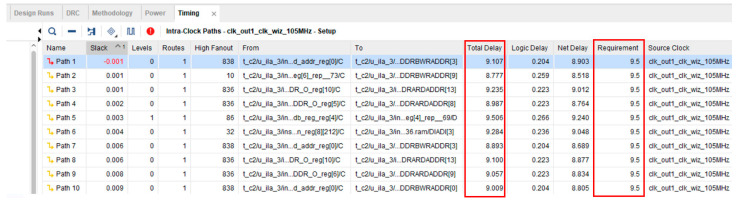
The timing report of the proposed CNN hardware.

To verify the result of the hardware implementation, after completing the Verilog RTL code of the CNN hardware design, the Vivado software developed by Xilinx was used to synthesize the Verilog code and the Virtex-7 VC707 evaluation board was used to implement the circuit. Figure 10 shows the timing report after circuit implementation. Moreover, the clock constraint during circuit implementation was 9.5 ns. The total delay of the critical path was smaller than 10 ns. Therefore, the proposed CNN hardware circuit can correctly operate at 100 MHz. Moreover, the power analysis report of Vivado showed that the power consumption was only 682 mW at a clock frequency of 100 MHz.

In the proposed CNN hardware design, the single-port ROM and RAM support the circuit operation. The block memory generator is used to generate the ROM or RAM. Although the maximum RAM unit is 36 Kbits, it can also be divided into two parts of RAM with 18 Kbits. Therefore, the minimum unit of ROM and RAM is 18 Kbits. By summing up all the memory blocks, the block memory generator must generate a total of 396 Kbits of memory, as shown in Table 14. However, the CNN hardware circuit uses only 280.17 Kbits of memory.

The test data were stored in the ROM of the FPGA to check the model accuracy of the proposed CNN hardware design on the FPGA. However, due to a large amount of test data, a ROM in Vivado was only sufficient for 655 test images with a size of 1 × 1600. Therefore, each label had 655 test images for accuracy evaluation in each test while programming the FPGA.

Table 15 shows the number of correctly predicted images after 12 tests. Each test set had 655 test images on each label. Moreover, these test images were randomly selected without repetition and stored in the ROM of an FPGA. The proposed CNN hardware circuit can operate at 100 MHz in these tests, which was also the fastest operating speed of the proposed design. Compared to the RTL-level CNN design, there was an accuracy loss of 1%. However, the accuracy loss was only 0.97% compared to that of the Python result.

Table 16 shows the corresponding accuracy at individual implementation stages. From the Tensorflow to the FPGA implementation, the accuracy loss was about 2.2%. Moreover, due to the hardware resource limitations of the FPGA, only 39% of the test images were randomly selected to test the correctness of the proposed CNN hardware circuit on FPGA.

Moreover, one of this work’s design goals is to achieve real-time response ability. Therefore, the following paragraphs investigate whether the proposed CNN hardware accelerator can perform real-time bearing fault diagnosis. First, Table 17 shows the clock cycles required for each layer calculation. According to the description of the PU dataset, the sampling rate of the current signal data was 64 K sample/s. Moreover, the proposed design used a down-sampling factor of 10; so, the sampling rate was reduced to 6.4 K sample/s, which means that the time to receive one sensor data point was 156,250 ns. Therefore, the waiting time for the first image was 250,000,000 ns, as shown in Equation (5).

(5) 250,000,000 ns=156,250 ns × 1600 

When the proposed CNN hardware circuit receives the first image, the data point of the sensor continues to be entered during the CNN hardware computation. However, the memory resource for the input image cannot be released until the CNN model calculation is complete. Therefore, an additional buffer is needed to store the sensor data before the previous image calculation is complete. In this paper, the highest clock frequency was 100 MHz, as shown in Figure 10. Therefore, 1600 data points of an input image require a total of 25,000,000 cycles, and 3,624,603 cycles are required for the CNN model calculation, as shown in Table 17. Therefore, approximately 232 data points for the next input image must be stored in an additional buffer, as shown in Equation (6). The additional 232 × 9-bit buffer is required for real-time fault diagnosis at the highest circuit speed. Therefore, when the CNN model calculation for the first image is complete, the proposed CNN hardware accelerator needs to wait for 1368 (= 1600 − 232) data points of the following input image. The proposed CNN hardware accelerator can then perform bearing fault diagnosis for the next input image. Finally, the proposed CNN hardware accelerator can process and analyze the data generated by the current sensor at a sampling rate of 64 K samples/s in real time to enable real-time bearing failure analysis.

(6) 3,624,603 × 10 ÷ 156,250 ≈ 232

In addition, the previous works did not specify the maximum time required for diagnostics to detect bearing faults after inputting the sensor data. In this paper, a down-sampling factor of 10 was used to increase the time length of the signal that can be viewed in an image and to diagnose the bearing condition more accurately. Therefore, the response time of the CNN circuit mainly involves the wait for the input of the sensor data. Thus, adding the time to receive the sensor data and the diagnostic time of the CNN hardware circuit, the response time of the proposed CNN hardware circuit was 0.28 s, as shown in Equation (7).

(7) 0.28 s=250,000,000 ns+3,624,603 cycles×10 ns

If a down-sampling factor of 1 is used as a compromise to reduce the response time, the model accuracy is reduced to about 96%. However, the response time can be reduced to 0.06 s, as shown in Equations (8) and (9).

(8) 25,000,000 ns=15,625 ns×1600

(9) 0.06 s=25,000,000 ns+3,624,603 cycles×10 ns

Betta [29] proposed a real-time fault detection system for rotating machinery to achieve good diagnostic performance. However, the time the system takes from measuring the signal to diagnosing the fault is 0.3 s. The proposed CNN hardware circuit can achieve a faster response time than Betta’s system [29]. Therefore, the proposed design can perform real-time fault diagnosis, prevent bearing damage, and extend the bearing’s lifetime.

## 5. Conclusions

To satisfy the requirement of real-time property in bearing fault diagnosis, this paper proposes a one-dimensional CNN architecture to reduce the response time. Furthermore, the proposed method can be further implemented in hardware and then installed on real machinery for real-time bearing fault diagnosis in any real-world machine tools using bearings. More specifically, the proposed design uses a current signal instead of the vibration signal, which reduces the cost of the vibration sensor and is a better solution for bearings that are difficult to disassemble. Furthermore, a new quaternary quantization method is proposed better to represent the weight values in the one-dimensional CNN model and to achieve better accuracy than the ternary quantization method. After training with the bearing data of PU, acceptable accuracy below the limited bit width was achieved by testing, and the hardware cost also did not become too large.

Moreover, the feasibility of realizing the proposed CNN model in hardware was investigated in this paper. The proposed CNN model was not only evaluated at the software stage but also implemented on an FPGA evaluation board to obtain classification accuracy at the hardware level and to analyze the resources required for the proposed design. In addition, this work also analyzed the real-time bearing fault diagnosis condition. As a result, at the fastest clock speed of the proposed CNN hardware design, only 2088 bits of the additional buffer were required. Finally, the accuracy of the proposed CNN hardware design implemented on FPGA was 96.37%, which was only a loss of 2.2% compared to the accuracy using Tensorflow.

On the other hand, in addition to the current data, the bearing data of PU also provides simultaneous measurement of vibration signals. Therefore, in the future, the authors will consider fault classification through multi-sensor fusion to build a neural network with better accuracy. Moreover, a tradeoff will exist between the accuracy and the hardware cost. Abhinav [30] proposes modular neural network architectures. In the future, the authors will also consider using a modular neural network architecture to reduce the CNN hardware accelerator’s model parameters and power consumption.

## Figures and Tables

**Table 1:** The setting of the hyperparameters in TensorFlow while adjusting the CNN model.

Hyperparameter	Setting
Epoch	300
Batch size	128
Learning rate	0.001

**Table 2:** Network setting label and the corresponding fault type.

Label	Fault Type	Data Amount
0	Healthy	6
1	Outer Race Fault (OR)	12
2	Inner Race Fault (IR)	9
3	Combined Outer and Inner Race Fault (CR)	5

**Table 3:** Ball-passing frequencies corresponding to two rotational speeds.

Ball-Passing Frequency	900 rpm	1500 rpm
*F_o_*	45.7 Hz (21 ms)	74.1 Hz (13 ms)
*F_i_*	76.3 Hz (13 ms)	123.6 Hz (8 ms)

**Table 4:** The comparison of CNN model accuracies with various input image sizes.

Image Size	The Observation Time of One Image	Accuracy
1 × 625	9.7 ms	87.6%
1 × 1024	16 ms	93.1%
**1 × 1600**	**25 ms**	**96.5%**
1 × 2500	39 ms	96.3%
1 × 3600	56 ms	95.7%

**Table 5:** The input and output image sizes of the proposed CNN.

Layer	Kernel Size	Input Image Size	Output Image Size
Conv1	1 × 7 × 12	1 × 1600	1 × 1600 × 12
Max pooling		1 × 1600 × 12	1 × 400 × 12
Conv2	1 × 7 × 32	1 × 400 × 12	1 × 400 × 32
Max pooling		1 × 400 × 32	1 × 100 × 32
Conv3	1 × 7 × 64	1 × 100 × 32	1 × 100 × 64
Max pooling		1 × 100 × 64	1 × 25 × 64
Conv4	1 × 7 × 64	1 × 25 × 64	1 × 25 × 64
Max pooling		1 × 25 × 64	1 × 7 × 64
Conv5	1 × 7 × 64	1 × 7 × 64	1 × 7 × 64
Max pooling		1 × 7 × 64	1 × 2 × 64
FC		1 × 128	1 × 4

**Table 6:** The comparison of the model accuracies with different quantizing weight methods.

Quantization Method	Weight	Threshold	Accuracy
None	Floating-point	-	99.3%
Binary [26]	{−1, 1}	0	74.2%
Ternary [27]	{−1, 0, 1}	±0.05	83.6%
Ternary Floating [28]	{−*W_n_*, 0, *W_p_*}	±0.05	90.8%

**Table 7:** The mean value of the weights of each layer before quantization.

Mean	Test 1	Test 2	Test 3	Test 4
Conv1 weight	0.066	−0.056	−0.121	−0.041
Conv2 weight	−0.064	0.034	−0.034	−0.094
Conv3 weight	−0.056	−0.078	−0.124	0.007
Conv4 weight	−0.111	−0.115	−0.012	−0.178
Conv5 weight	−0.096	0.111	−0.078	0.074

**Table 8:** The comparison of accuracies obtained by different settings of output channels number.

Version	Output Channels in Each Layer					Param #	Accuracy
	C_1_	C_2_	C_3_	C_4_	C_5_		
1	8	32	32	64	64	52 K	88.6%
2	8	32	32	64	128	82 K	91.8%
3	12	16	32	64	128	78 K	93.7%
4	12	16	64	64	64	66 K	96.4%
5	12	32	32	64	128	83 K	97.6%
**6**	**12**	**32**	**64**	**64**	**64**	**75 K**	**98.5%**
7	16	32	64	64	64	76 K	98.7%

**Table 9:** The comparison of accuracies with different settings of total bits of one weight in the lookup table.

Kernel Table Bits			Accuracy
Total Bits	Integer Bits	Decimal Bits	
11	5	6	88.71%
12	5	7	93.2%
13	5	8	97.48%
**14**	**5**	**9**	**98.08%**
15	5	10	98.25%

**Table 10:** The comparison of model accuracies with different settings of bit number of the feature map.

Bits of One Feature Map Value			Accuracy
Total Bits	Integer Bits	Decimal Bits	
13	8	5	92.1%
14	8	6	95.22%
15	8	7	96.62%
**16**	**8**	**8**	**97.35%**
17	8	9	97.56%

**Table 11:** The comparison of the memory usage after using fixed-point parameters.

Memory Type	Memory	Total Bits before the Fixed Point	Total Bits after the Fixed Point	Reduction Ratio
ROM	Conv_Weight	2,382,464	148,904	93.75%
	FC_Weight	16,384	1024	93.75%
	BN	15,104	8968	40.62%
RAM	Fmap_1	153,600	76,800	50%
	Fmap_2	102,400	51,200	50%
The sum of all bits		2,669,952	286,896	89.25%

**Table 12:** The comparison of accuracies of each category using Python and RTL codes.

Fault Type	Label	Python	RTL
Healthy	0	97.49%	97.97%
OR Fault	1	99.79%	99.27%
IR Fault	2	95.69%	95.28%
CR Fault	3	96.39%	96.86%
Total	-	97.34%	97.53%

**Table 13:** The comparison of the proposed method to related works at the software level.

	[1] IEEE Trans. Meas. ‘20		[12] IEEE Access ‘20	[13] INISTA ‘20	[14] Sensors ‘19	Proposed Work
Dataset	PU		PU	PU	PU	PU
Signal type	Vibration	Current	Current	Current	Current	Current
Architecture	2-D CNN + MLP		2-D Residual CNN	1-D CNN + LSTM + KNN	1-D CNN + SVM	1-D CNN
Datapre-processing	Gray image		Normalize + overlap + gray image	None	Overlap + FFT + normalize	Reduce excess bits + overlap
Type of classification	Fault location (3 types without IR + OR combined fault)		Fault location (3 types without IR + OR combined fault)	Fault location (3 types without IR + OR combined fault)	Fault location (3 types without IR + OR combined fault)	Fault location (4 types)
Image size	80 × 80		224 × 224	1 × 6400	N/A	1 × 1600
Accuracy	99.4%	98.3%	98.7%	88.8~98.93%	98.17%	98.58%

**Table 14:** Size and composition of each memory block.

Memory Name	18 Kbits RAM	36 Kbits RAM	Total Kbits
Input_image_ROM	1	0	18
Conv_Weight_ROM	0	5	180
FC_Weight_ROM	1	0	18
BN_ROM	1	0	18
Fmap_1	1	2	90
Fmap_2	0	2	72
Total	4	9	396

**Table 15:** Accuracy of the CNN hardware in multiple tests on an FPGA.

Number of Correct	Label 0	Label 1	Label 2	Label 3
Test set 1	642	652	625	637
Test set 2	644	644	615	634
Test set 3	637	643	614	632
Test set 4	633	647	622	641
Test set 5	623	648	619	635
Test set 6	631	643	623	624
Test set 7	628	640	627	616
Test set 8	630	652	605	627
Test set 9	615	645	632	630
Test set 10	627	638	621	636

**Table 16:** Corresponding test accuracy at each implementation stage.

Each Stage Operation	Accuracy
Input data fixed-point training (9 bits)	98.58%
BN parameters fixed-point (19 bits)	98.3%
Weight value fixed-point (14 bits)	98.08%
Feature map fixed-point (16 bits)	97.34%
RTL code	97.53%
FPGA implementation	96.37%

**Table 17:** The number of cycles used in each layer calculation.

	# Cycles
Layer 1	134,489
Layer 2	1,077,519
Layer 3	1,445,905
Layer 4	741,393
Layer 5 + FC	225,297
Total	3,624,603

## Data Availability

Not applicable.

## Glossary

***Term***	**Definition**
*d*	Ball diameter
*D*	Pitch diameter
$F_{i}$	Inner race ball-passing frequency
$F_{o}$	Outer race ball-passing frequency
$F_{r}$	Rotary frequency
*n*	Number of rolling elements
$W_{n}$	Quantization factors for negative weight
$W_{P}$	Quantization factor for positive weight
$w^{t}$	Quantized ternary weights
*x*	A variable for threshold adjustment in equations
α	Bearing contact angle between the ball and the cage
*β*	Shift parameter for batch normalization
*γ*	Scale parameter for batch normalization
σ	Standard deviation of the weights in each layer
